# Physical activity and optimal self-rated health of adults with and without diabetes

**DOI:** 10.1186/1471-2458-10-365

**Published:** 2010-06-23

**Authors:** James Tsai, Earl S Ford, Chaoyang Li, Guixiang Zhao, Lina S Balluz

**Affiliations:** 1 Division of Adult and Community Health, National Center for Chronic Disease Prevention and Health Promotion Centers for Disease Control and Prevention (CDC), Atlanta, GA 30341, USA

## Abstract

**Background:**

Regular physical activity can improve people's overall health and contribute to both primary and secondary prevention of many chronic diseases and conditions including diabetes. The aim of this study was to examine the association between levels of physical activity and optimal self-rated health (SRH) of U.S. adults with and without diabetes in all 50 states and territories of the Unites States.

**Methods:**

We estimated the prevalence of optimal SRH by diabetes status of 430,912 adults aged 18 years and older who participated in the 2007 state-based survey of the Behavioral Risk Factor Surveillance System (BRFSS). Prevalence ratios were produced with multivariate Cox regression models using levels of physical activity as a predictor and status of optimal SRH as an outcome variable while controlling for sociodemographic and behavioral health risk factors.

**Results:**

The prevalence of reporting optimal SRH was 53.3%, 52.2%, and 86.2% for adults with type 1 diabetes, type 2 diabetes, and without diabetes, respectively. Also in the aforementioned order, adults who reported being active had an increased likelihood of 81%, 32%, and 18% for reporting optimal SRH, when compared with adults who reported being inactive.

**Conclusions:**

Regular physical activity of adults, particularly adults with diabetes, is associated with optimal SRH. The findings of this study underscore the importance of advising and motivating adults with diabetes so that physical activity can be integrated into their lifestyle for diabetes care. Additionally, a population-based effort to promote physical activity in communities may benefit adults in general by improving their overall health and well-being.

## Background

Regular physical activity can improve people's overall health and reduce various risks for morbidity and mortality due to a sedentary lifestyle [1,2]. Accumulating evidence from observational and experimental studies shows that routine physical activity exerts enormous benefits for both primary and secondary prevention of diabetes mellitus, cardiovascular disease, obesity, cancer, musculoskeletal diseases, and depression [3-8]. For most health outcomes, the report of *2008 Physical Activity Guidelines for Americans *(2008 Guidelines) recommends a combination of active time equivalent to at least 150 minutes of moderate activity a week. These health benefits can occur in healthy people and in individuals who currently have or are at risk of developing chronic diseases and conditions [1,3,9]. In particular, regular physical activity may improve glucose homeostasis and insulin sensitivity thereby preventing, delaying or reversing the development of type 2 diabetes [3,4,7,10].

Self-rated health (SRH) is a powerful indicator of a population's overall well-being because its lower ratings (i.e., fair or poor) are strongly predictive of future morbidity, mortality, functional decline, and health care utilization [11-16]. For instance, individuals with ''poor'' SRH had a two-fold higher mortality risk than those with ''excellent'' SRH [14]. In addition, the lower ratings of diabetic patients have been linked to diabetes-related complications such as lower extremity amputation, blindness, kidney failure, and cardiovascular diseases (e.g., heart disease and stroke) [17]. Not surprisingly, maintaining and improving health-related quality of life of people with diabetes is a public health goal, whereas assisting patients to achieve optimal SRH is a quality indicator in primary care [17-19].

Despite substantial practical interest to consumers, researchers, and practitioners, much remains unknown regarding levels of physical activity in relation to optimal SRH pertaining to diabetes in adults in the general population. The aim of this study was to examine the association between levels of physical activity and optimal SRH of adults with and without diabetes in the United States by using the data from the 2007 state-based survey of the Behavioral Risk Factor Surveillance System (BRFSS).

## Methods

### Participants

The Behavioral Risk Factor Surveillance System (BRFSS) is the world's largest, ongoing, state-based, random-digit-dialed telephone survey that collects information on health risk behaviors, preventive health practices, and health care access primarily related to chronic disease and injury, and is considered to be exempt from review by the Centers for Disease Control and Prevention's Institutional Review Board [20]. Details about its purpose, sampling methods, data collection, and reporting are available elsewhere [20]. Survey information from non-institutionalized adults aged 18 years and older has been used to track health conditions and risk behaviors for improving the health of the American people. With a survey median cooperation rate of 72.1% in 2007, a total of 430,912 individuals from all 50 states, the District of Columbia, Guam, Puerto Rico, and the Virgin Islands participated in the survey [20]. Based on the history of diabetes collected (Table 1), participants were considered to have type 1 diabetes if their age at diagnosis was less than 30 years and they were currently using insulin. Participants were considered to have type 2 diabetes if their age at diagnosis was at least 30 years or if their age at diagnosis was less than 30 years and they were currently not using insulin [21-23].

**Table 1:** Questions about physical activity, self-rated health, and medical history for the year 2007, BRFSS, United States

Item	Survey Questions
**Physical activity**	
	Moderate activity
	(1) When you are not working, in a usual week, do you do moderate activities for at least 10 minutes at a time, such as brisk walking, bicycling, vacuuming, gardening, or anything else that causes some increase in breathing or heart rate?
	(2) How many days per week do you do these moderate activities for at least 10 minutes at a time?
	(3) On days when you do moderate activities for at least 10 minutes at a time, how much total time per day do you spend doing these activities?
	
	Vigorous activity
	(1) When you are not working, in a usual week, do you do vigorous activities for at least 10 minutes at a time, such as running, aerobics, heavy yard work, or anything else that causes large increases in breathing or heart rate?
	(2) How many days per week do you do these vigorous activities for at least 10 minutes at a time?
	(3) On days when you do vigorous activities for at least 10 minutes at a time, how much total time per day do you spend doing these activities?
	
**Self-rated health**	
	Would you say that in general your health is excellent, very good, good, fair, or poor?
**Medical history**	
	Diabetes
	(1) Have you ever been told by a doctor that you have diabetes?
	(2) How old were you when you were told you have diabetes?
	(3) Are you now taking insulin?
	
	Cardiovascular diseases
	Have you ever been told by a doctor, nurse, or other health professional that you have had:
	(1) a heart attack, also called a myocardial infarction;
	(2) angina or coronary heart disease;
	(3) a stroke?
	
	Arthritis
	Have you ever been told by a doctor or other health professional that you have some form of arthritis, rheumatoid arthritis, gout, lupus, or fibromyalgia?
	
	Disability
	(1) Are you limited in any way in any activities because of physical, mental, or emotional problems?
	(2) Do you now have any health problem that requires you to use special equipment, such as a cane, a wheelchair, a special bed, or a special telephone?

### Measures

#### Physical activity

To assess participation in moderate and vigorous activities, participants were asked a series of questions listed in Table 1. According to the 2008 Guidelines, one minute of vigorous-intensity activity was considered equivalent to 2 minutes of moderate-intensity activity [1]. Thus, participants were classified as being active if they reported at least 150 minutes per week of moderate activity, or at least 75 minutes per week of vigorous activity, or a combination of moderate and vigorous activity totaling at least 150 minutes per week. Participants were classified as insufficiently active if they reported moderate or vigorous activity in episodes of at least 10 minutes, but did not accrue a combination of time equivalent to 150 minutes of moderate activity per week. Participants were classified as being inactive if they reported no moderate or vigorous activity in episodes of at least 10 minutes per episode.

#### Optimal SRH

SRH is a brief but valid proxy measure for overall health status [11,13,14]. As part of the health-related quality of life questions, the BRFSS survey asked the participants to rate their overall health (Table 1). We dichotomized these responses into the categories of optimal (i.e., excellent, very good, or good) and suboptimal (i.e., fair or poor) for this analysis.

#### Behavioral Health Risk Factors

In addition to the sociodemographic variables such as age, sex, race/ethnicity, education, marital status, and employment, information about behavioral health risk factors also was collected and defined for the analysis. For instance, based on the responses to the questions "Have you smoked at least 100 cigarettes in your entire life?" and "Do you now smoke cigarettes every day, some days, or not at all?", current smokers were defined as those who had smoked 100 cigarettes or more during their lifetime and who currently smoked every day or some days. Similarly, participants who responded affirmatively to the question "During the past 30 days, have you had at least one drink of any alcoholic beverage such as beer, wine, a malt beverage or liquor?" were considered current drinkers. Participants who responded affirmatively to the questions about arthritis or disability were considered to have arthritis or disability (Table 1). Additionally, participants who responded affirmatively to any of the three questions about cardiovascular diseases (CVDs) were considered to have a history of CVDs (Table 1). Participants who responded affirmatively to the question "Do you have any kind of health care coverage, including health insurance, prepaid plans such as HMOs, or government plans such as Medicare?" were considered to have health care access. Additionally, body mass index (BMI), the self-reported weight (kilograms) divided by the square of height (meters), was categorized as (1) neither overweight nor obese (< 25 kg/m^2^), (2) overweight (25 - < 30 kg/m^2^), and (3) obese (≥ 30 kg/m^2^). To assess diabetes education among participants with diabetes, the responses to the question "Have you ever taken a course or class in how to manage your diabetes yourself?" were evaluated.

### Statistical Analysis

We estimated the prevalence of optimal SRH among participants with and without diabetes and subgroups with regard to age, sex, race/ethnicity, education, employment, marital status, smoking, drinking, leisure time physical activity, history of cardiovascular diseases and arthritis, disability, BMI, health care access, and levels of physical activity [24]. Because optimal SRH is a common health outcome in the study population, logistic regression models may not produce an accurate approximation [25,26]. Therefore, to examine the association between levels of physical activity and optimal SRH, prevalence ratios (PR) and 95% confidence intervals were generated with multivariate Cox regression analyses for complex samples with constant time using levels of physical activity as a predictor and status of optimal SRH as an outcome variable. Cox regression models have shown PR similar to those of using Log-binomial regression models [25,26]. We present one unadjusted and two adjusted models controlling for sociodemographic characteristics such as age, sex, race/ethnicity, education, employment, and marital status (model 1), as well as additional covariates such as smoking, drinking, health care access, disability, leisure time physical activity, BMI, history of cardiovascular diseases and arthritis (model 2). For participants who reported being active with optimal SRH, we calculated the number of days per week they had engaged in moderate or vigorous activity or a combination of both types of physical activity. Moreover, the estimated proportion for levels of physical activity of adults with diabetes was calculated according to their status of ever having taken a course or class for managing diabetes.

We performed the analysis using SPSS 17 Complex Samples for Survey Analysis (*SPSS Inc., Chicago, IL, 2008*) to account for multiple stages of sampling, stratification, and clustering [27]. To obtain appropriate statistics, all analyses in this study were weighted according to the standard procedures for analyzing sample survey data [28]. Variance estimates were approximated based on Taylor series linearization [27].

## Results

The unadjusted prevalence of reporting optimal SRH was 53.3%, 52.2%, and 86.2%, among participants with type 1 diabetes, type 2 diabetes, and without diabetes, respectively (Table 2). Regardless of status of diabetes, the subgroup analysis showed that a greater prevalence of optimal SRH was found in participants who were currently employed, who had no history of cardiovascular diseases or arthritis, who had no disability, or who were neither overweight nor obese, when compared with their respective counterparts (*p *< 0.001 for χ^2 ^test). In addition, a higher prevalence of optimal SRH was found in participants who were aged 18-44 years with type 1 diabetes or without diabetes, participants with type 2 diabetes or without diabetes who were male, who were non-Hispanic White, who had a college education, who were married, or who were not current smokers, who were current drinkers, or who had access to health care, as compared with their respective counterparts (*p *< 0.001 for χ^2 ^test).

**Table 2:** Prevalence of optimal SRH by socio-demographic characteristics and behavioral health risk factors among adults with and without diabetes for the year 2007, BRFSS, United States

	Optimal self-rated health ^a^					
	**Type 1 diabetes**^b^ (n ^d ^= 1,376)		**Type 2 diabetes**^c^ (n = 29,572)		Without diabetes (n = 380,757)	
	%	**95% CI **^e^	%	95% CI	%	95% CI
**Overall**	53.3	47.5-59.1	52.2	51.2-53.8	86.2	85.9-86.4
**Age (years)**						
18-24	86.2	69.2-94.6	41.1	19.0-67.4	90.6	89.5-91.5
25-34	65.0	53.2-75.2	63.2	52.2-73.0	90.0	89.2-90.6
35-44	47.9	38.1-57.8	57.5	52.0-62.8	89.4	88.9-89.9
45-64	39.5	31.3-48.4	50.8	49.0-52.7	84.5	84.1-84.8
≥65	43.0	30.3-56.8	52.6	50.7-54.5	76.5	76.0-77.0
**Sex**						
Male	56.8	47.7-65.5	55.2	53.3-57.2	86.8	86.4-87.3
Female	49.3	42.4-56.2	49.6	47.9-51.3	85.5	85.2-85.9
**Race/ethnicity**						
Non-Hispanic White	59.4	53.2-65.3	57.1	55.8-58.3	88.7	88.5-88.9
Non-Hispanic Black	38.8	25.8-53.6	48.9	45.2-52.5	83.3	82.4-84.2
Hispanic	46.7	22.3-72.8	32.8	28.3-37.6	76.0	74.7-77.2
Other	42.3	24.8-62.0	58.4	51.9-64.7	87.1	86.0-88.2
**Education**						
Less than high school	44.8	20.8-71.6	28.8	25.7-32.0	65.6	64.3-66.9
High school graduates	41.7	31.9-52.3	49.0	47.1-50.9	83.1	82.6-83.6
College education	61.3	54.5-67.7	63.6	61.7-65.3	91.3	91.1-91.6
**Employment**						
Employed	66.5	58.9-73.3	67.0	64.5-69.4	91.5	91.2-91.8
Unemployed	55.3	34.4-74.5	44.8	37.4-52.3	79.6	78.1-81.1
Not in workforce	36.3	26.1-47.8	44.5	43.0-46.0	76.8	76.3-77.2
**Marital Status**						
Married ^f^	53.4	46.4-60.3	55.7	53.9-57.4	87.9	87.6-88.2
Unmarried ^g^	53.1	43.4-62.6	46.8	45.0-48.7	83.0	82.5-83.5
**Current smoker**						
Yes	42.6	32.1-53.9	43.4	40.4-46.5	80.0	79.4-80.7
No	56.0	49.3-62.6	54.1	52.6-55.5	87.7	87.4-88.0
**Current drinker**						
Yes	65.5	56.9-73.1	64.1	61.6-66.4	90.4	90.1-90.8
No	45.0	36.9-53.4	47.5	46.0-49.0	81.1	80.7-81.5
**History of cardiovascular diseases**						
Yes	34.3	23.4-47.1	35.8	33.3-38.4	57.0	55.9-58.1
No	59.1	52.3-65.5	59.2	57.7-60.8	88.4	88.1-88.6
**History of arthritis**						
Yes	34.1	26.5-42.8	44.4	42.8-46.0	74.1	73.6-74.6
No	61.2	54.2-67.8	61.2	59.1-63.2	90.2	89.9-90.5
**Disability**						
Yes	21.4	15.6-28.7	32.7	31.1-34.5	59.9	59.2-60.6
No	72.6	65.4-78.9	68.0	66.1-69.8	92.0	91.8-92.3
**Have health care access**						
Yes	55.1	48.9-61.2	53.9	52.5-55.2	87.4	87.2-87.7
No	37.8	23.1-55.3	39.7	35.4-44.2	79.4	78.5-80.3
**Body mass index (kg/m^2^)**						
Neither overweight nor obese (< 25)	61.8	50.0-72.3	53.9	50.0-57.7	88.9	88.5-89.3
Overweight (25 -< 30)	60.4	50.8-69.3	57.0	54.6-59.2	87.6	87.2-88.0
Obese (≥ 30)	34.6	27.8-42.5	49.7	47.9-51.5	80.6	79.9-81.2

a. Self-rated health reported as "excellent", "very good", or "good".

b. Age at diagnosis < 30 years and currently using insulin.

c. Age at diagnosis ≥30 years. Or, age at diagnosis < 30 years and currently not using insulin.

d. Maximum subgroup sample size.

e. Confidence interval.

f. Included unmarried couples.

g. Reported as "divorced", "widowed", "separated", or "never married".

With an increased level of physical activity associated with a greater prevalence of reporting optimal SRH, participants who reported being active or insufficiently active had a higher prevalence of optimal SRH than adults who reported being inactive, regardless of diabetes status (Table 3). Upon controlling for covariates such as age, sex, race/ethnicity, education, employment, marital status, smoking, drinking, BMI, health care access, disability, history of cardiovascular diseases and arthritis in Cox regression models, participants who reported being active or insufficiently active were more likely to indicate optimal SRH (i.e., PR = 1.81-1.86 for type 1 diabetes; or PR = 1.24-1.32 for type 2 diabetes; PR = 1.13-1.18 for without diabetes), when compared with adults who reported being inactive (Table 3).

**Table 3:** Estimated prevalence and prevalence ratios for reporting optimal SRH by levels of physical activity among adults with and without diabetes for the year 2007, BRFSS, United States

Levels of physical activity by status of diabetes	Outcome							
	
	**Optimal SRH**^a^		Un-adjusted		**Adjusted Model 1**^b^		**Adjusted model 2**^c^	
	%	**95% CI**^d^	Prevalence ratio	95% CI	Prevalence ratio	95% CI	Prevalence ratio	95% CI
**Type 1 diabetes **^e ^(n = 1,274) ^f^								
Inactive	22.0	12.2-36.5	1.00	Reference	1.00	Reference	1.00	Reference
Insufficiently active ^g^	50.5	41.0-60.0	2.29	1.28-4.13	1.75	1.21-3.45	1.86	1.19-2.92
Active ^h^	63.1	55.3-70.2	2.87	1.62-5.06	1.92	1.10-2.81	1.81	1.18-2.77
**Type 2 diabetes **^i ^(n = 27,257)								
Inactive	34.7	32.3-37.2	1.00	Reference	1.00	Reference	1.00	Reference
Insufficiently active	52.1	49.7-54.5	1.50	1.38-1.63	1.36	1.26-1.47	1.24	1.14-1.34
Active	62.1	60.0-64.1	1.79	1.65-1.93	1.56	1.45-1.68	1.32	1.22-1.42
**Without diabetes **(n = 353,928)								
Inactive	67.1	66.1-68.1	1.00	Reference	1.00	Reference	1.00	Reference
Insufficiently active	84.2	83.5-84.8	1.25	1.23-1.28	1.18	1.16-1.20	1.13	1.11-1.15
Active	90.9	90.6-91.1	1.35	1.33-1.37	1.26	1.24-1.28	1.18	1.16-1.20

a. Self-rated health reported as "excellent", "very good", or "good".

b. Adjusted for age, sex, race/ethnicity, education, employment, and marital status.

c. Adjusted for age, sex, race/ethnicity, education, employment, marital status, smoking, drinking, BMI, health care access, disability, history of any cardiovascular diseases (i.e., heart attack, angina, coronary heart disease, or stoke) and arthritis.

d. Confidence interval.

e. Age at diagnosis < 30 years and currently using insulin.

f. Maximum subgroup sample size.

g. Reported moderate or vigorous activity in episodes of at least 10 minutes, but did not accrue a combination of time equivalent to 150 minutes of moderate activity per week.

h. At least 150 minutes of moderate physical activity per week; or 75 minutes of vigorous physical activity per week; or an equivalent combination of moderate and vigorous physical activity.

i. Age at diagnosis ≥30 years. Or, age at diagnosis < 30 years and currently not using insulin.

**Figure 1:**
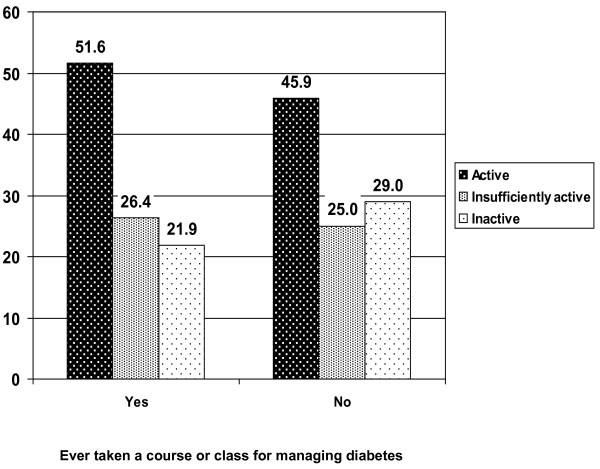
**Estimated proportions for physical activity levels by status of ever taken a course or class for managing diabetes among adults with diabetes for the year 2007, BRFSS, United States**.

Of the participants who reported being active with optimal SRH, about 90% reported engaging in moderate or vigorous activity or a combination of both types of physical activity for at least 5 days a week, regardless of status of diabetes (Table 4). Additionally, adults with diabetes who had ever taken a course or class (55.4%) for managing diabetes had a higher proportion of being active than diabetic patients without taking such a class (*p *< 0.001 for χ^2 ^test) (Figure 1).

**Table 4:** Number of days per week for engaging in moderate or vigorous activity or a combination of both types of physical activity among adults who reported being active with optimal SRH for the year 2007, BRFSS, United States

	**Number of days per week for engaging in physical activity (n = 140,762)**					
	
	**1-2 days**		**3-4 days**		**≥5 days**	
	**%**	**CI**^a^	**%**	**CI**	**%**	**CI**
	
**Diabetes status**						
**Type 1 diabetes**	1.1	0.2-5.2	5.2	2.8-9.3	93.7	89.0-96.5
**Type 2 diabetes**	0.8	0.5-1.4	8.6	7.0-10.4	90.6	88.7-92.2
**Without diabetes**	0.8	0.7-0.9	7.8	7.5-8.2	91.4	91.1-91.7

a. 95% confidence interval.

b. Chi-square test (*p *> 0.05).

## Discussion

SRH is an indicator of chronic disease that cuts across the Healthy People 2010 objectives; it is also a well-established quality of life measure for public health research and practice [19,29]. The two overarching goals of *Healthy People 2010 *are (1) to help people of all ages increase life expectancy and improve their quality of life, and (2) to eliminate health disparities among different segments of the population [19,29]. Attaining these goals is especially challenging for individuals with diabetes because of disease severity, complications, and comorbid conditions. Health care providers are influential in helping people with diabetes to feel overall healthy as the quality of life of such individuals is often greatly impaired.

Although SRH ratings are generated by individuals through a subjective and contextual process [13], research shows that biological, physiological, and psychological factors are major determinants of SRH [13,30-32]. To date, there is overwhelming evidence that regular physical activity affects nearly all systems in the body and leads to many favorable chronic adaptations and acute changes [3]. Physical activity can bring about numerous health benefits which may include but are not limited to improving cardio-respiratory functions, body composition, lipid lipoprotein profiles, glycemic control, as well as reducing blood pressure, systemic inflammation, stress, anxiety and depression [3,7].

Our findings provide evidence that regular physical activity is associated with better SRH among adults. Although the prevalence of optimal SRH was substantially lower for adults with diabetes than for adults without diabetes, adults with either type 1 or type 2 diabetes who engaged in regular physical activity were even more likely to report optimal SRH than adults without diabetes. Indeed, research demonstrates that regular physical activity may reduce the risk of developing diabetes in individuals who are at an increased risk of developing this condition [3,33-35]. However, when compared with adults who reported being inactive, adults with type 1 diabetes, type 2 diabetes, or without diabetes were more likely to report optimal SRH. Given that adults with diabetes are less likely to participate in physical activity at the recommended levels than adults without diabetes [36], diabetic adults who are currently inactive are strongly advised to engage in a level of physical activity as their abilities and conditions allow, even if this initial level does not meet the 2008 Guidelines.

Available evidence has shown that physical activity is safe when adults with chronic diseases perform according to their abilities [1]. Currently, the American Diabetes Association (ADA) recommends not omitting aerobic physical activity for more than 2 consecutive days, as the effect of a single instance of aerobic activity on insulin sensitivity lasts no more than 72 hours [37]. Our study indicated that a majority of adults who were active and who had optimal health status engaged in moderate or vigorous activity or a combination of both types of physical activity with a frequency of at least 5 days a week, regardless of their diabetes status. In addition, the ADA suggests that all levels of physical activity can be performed by people with type 1 diabetes who do not have complications and who have optimal blood glucose control [33,37]. A growing body of evidence has demonstrated the effectiveness of counseling in motivating and promoting physical activity in people with Type 2 diabetes [34]. However, adults with diabetes should consult with their health care providers to design a physical activity program specific to their needs [10,38,39]. Although regular physical activity is recognized as a cornerstone in diabetes care, it is often underused in health care practice [34]. In this study, a higher percentage of adults with diabetes who had ever taken a course or class for managing diabetes was physically active than adults who did not take such lessons. Hence, diabetes educators, as well as physicians and exercise therapists, are in a unique and influential position to advise and motivate adults with diabetes so that physical activity can be integrated into their lives thus optimizing their health and well-being.

The findings of our study are generally consistent with the 2008 Guidelines and several previous studies that demonstrated a graded relationship between levels of physical activity and SRH [40-44]. That is, an increased level of physical activity is associated with a greater likelihood of reporting optimal SRH. To our knowledge, such an analysis specific to diabetes has not been reported in the past. Although the BRFSS data have been found to provide valid and reliable estimates as compared with the national household surveys [45,46], our study has several limitations. For example, cross-sectional surveys such as BRFSS are not designed to determine a causal relationship. It was difficult to distinguish cause and effect for adults with diabetes, because physical activity could be a marker of well-being rather than the cause of healthier life. Without a doubt, there are many long-term complications of diabetes such as peripheral neuropathy, CVDs, blindness, peripheral vascular disease, and amputations that could interfere with the capacity for physical activity. Also, BRFSS is a landline survey, so people with no telephone or with cell phone only were excluded, possibly resulting in sampling bias. In addition, the survey used for this study was based on self-reported data. Studies have shown that self-reported data, particularly of less socially desirable behaviors, are subject to limitations of underreporting and recall bias [47]. For instance, self-reported physical activity is a subjective method of assessment; it may yield higher estimates of activity than the objective measurements obtain with an accelerometer and may not accurately reflect total energy expenditure of participants [48,49]. However, the BRFSS uses questions similar to the National Health Interview Survey for a number of measures. Measures for behaviors (e.g., physical activity), medical conditions, and health status are known to have moderate to high reliability and validity. The overall differences between these surveys ranged from 0.4 to 3.0 percentage points [45,46]. Moreover, our efforts at controlling potential confounders were limited by the availability of appropriate variables in the survey. As such, we were unable to control for behavioral health factors such as diabetes management including diet and medication, co-morbid disorders, as well as disease severity, or for diabetes-related physical and psychological barriers.

Compelling evidence from past research and from this study indicates that regular physical activity is a key strategy in the prevention of diabetes and is an essential component of diabetes care. Intervention from the health professionals involved in diabetes care is an important part of the solution to the societal problem of inactivity. To increase physical activity of all adults, *the Guide to Community Preventive Services *has identified several evidence-based approaches that can effectively increase physical activity at the community level [50], including campaigns and informational, behavioral and social, as well as environmental and policy approaches [50].

## Conclusions

Regular physical activity of adults, particularly adults with diabetes, is associated with optimal SRH. The findings of this study support the need to advise and motivate adults with diabetes so that physical activity can be integrated into their lifestyle for diabetes care. Additionally, a population-based effort to promote physical activity in communities may benefit adults in general by improving their overall health and well-being.

## Competing interests

The authors declare that they have no competing interests.

## Authors' contributions

JT and EF conceived of the study. All authors participated in the design of the study and data statistical analysis, and helped to draft the manuscript. All authors read and approved the final manuscript.

## Pre-publication history

The pre-publication history for this paper can be accessed here:

http://www.biomedcentral.com/1471-2458/10/365/prepub
